# Obesity in Children and Adolescents during COVID-19 Pandemic

**DOI:** 10.3390/children8020135

**Published:** 2021-02-12

**Authors:** Androniki Stavridou, Evangelia Kapsali, Eleni Panagouli, Athanasios Thirios, Konstantinos Polychronis, Flora Bacopoulou, Theodora Psaltopoulou, Maria Tsolia, Theodoros N. Sergentanis, Artemis Tsitsika

**Affiliations:** 1 2nd Department of Pediatrics, “P. & A. Kyriakou” Children’s Hospital, School of Medicine, National and Kapodistrian University of Athens, 115 27 Athens, Greece; stavroniki@hotmail.com (A.S.); kapsali.lila@gmail.com (E.K.); elenpana@med.uoa.gr (E.P.); athirios@med.uoa.gr (A.T.); kostas_arb@yahoo.gr (K.P.); tpsaltop@med.uoa.gr (T.P.); mariantsolia@gmail.com (M.T.); tsergentanis@yahoo.gr (T.N.S.); 2 Center for Adolescent Medicine and UNESCO Chair Adolescent Health Care, First Department of Pediatrics, “Agia Sophia” Children’s Hospital, School of Medicine, National and Kapodistrian University of Athens, 115 27 Athens, Greece; bacopouf@hotmail.com; 3 Department of Clinical Therapeutics, “Alexandra” Hospital, School of Medicine, National and Kapodistrian, University of Athens, 115 28 Athens, Greece

**Keywords:** COVID-19, obesity, weight gain

## Abstract

Background: The COVID-19 pandemic has led to special circumstances and changes to everyday life due to the worldwide measures that were imposed such as lockdowns. This review aims to evaluate obesity in children, adolescents and young adults during the COVID-19 pandemic. Methods: A literature search was conducted to evaluate pertinent studies up to 10 November 2020. Results: A total of 15 articles were eligible; 9 identified 17,028,111 children, adolescents and young adults from 5–25 years old, 5 pertained to studies with an age admixture (*n* = 20,521) and one study included parents with children 5–18 years old (*n* = 584). During the COVID-19 era, children, adolescents and young adults gained weight. Changes in dietary behaviors, increased food intake and unhealthy food choices including potatoes, meat and sugary drinks were noted during the ongoing COVID-19 pandemic. Food insecurity associated with financial reasons represents another concern. Moreover, as the restrictions imposed reduced movements out of the house, physical activity was limited, representing another risk factor for weight gain. Conclusions: COVID-19 restrictions disrupted the everyday routine of children, adolescents and young adults and elicited changes in their eating behaviors and physical activity. To protect them, health care providers should highlight the risk of obesity and provide prevention strategies, ensuring also parental participation. Worldwide policies, guidelines and precautionary measures should ideally be established.

## 1. Introduction

Coronavirus disease 2019 (COVID-19) is a multisystem disease caused by the severe acute respiratory syndrome coronavirus-2 (SARS-CoV-2), displaying high morbidity and mortality [1]. After more than 1,700,000 confirmed human cases and 111,600 deaths which had been reported in more than 200 countries [2], it was characterized by the WHO as pandemic on March 11, 2020 [3].

In response to COVID-19, multinational measures were implemented by the authorities, including school closures, lockdown, quarantine and social distancing recommendations, aiming at the mitigation of the virus spread as well as a decrease in the pressure on health care systems [4]. As a result, more than 2.6 billion people were subjected to home confinement [5]. Those circumstances led people to change their lifestyle and eating behaviors, including buying and consuming large quantities of preserved and processed food due to fear of food shortage. In parallel, the increase in sedentary behavior and screen time, as well as the decrease in physical activity, could also be associated with obesity [6]. In line with the above, the WHO estimated that the health of more than 1.9 billion overweight people (over 18 years) and 650 million obese people [7] will deteriorate due to stay-at-home orders. The newly developed term “covibesity” has been introduced to portray the aggravation in obesity rates due to the lockdown imposed during the pandemic [5].

As reported in the past, children and adolescents tend to gain weight during summer holidays [8], and it has been postulated that childhood obesity rates might increase proportionately to the number of months that schools remain closed [8]. Reports from the US stated that 1.27 million new childhood obesity cases were recorded until December 2020 if schools did not reopen [8]. Therefore, the need to promote the adoption of healthy nutritional status in children and adolescents during the COVID-19 pandemic has been actively supported [9].

On the other hand, obesity is considered to be one of the most prominent risk factors of severe COVID-19, increasing disease mortality, even in childhood [9]. In general, obesity and weight gain are associated with metabolic changes that increase the risk of non-communicable diseases, such as diabetes and cardiovascular disease [5,10]. 

Considering the special circumstances derived from COVID-19, including home confinement and prevention measures imposed worldwide, the research of the impact of weight gain and the possible increase in the prevalence of childhood and adolescent obesity rates is highly recommended. Therefore, this review aims to analyze changes in obesity and weight gain among children, adolescents (11–21 years) and young adults (up to 24 years) from the COVID-19 outbreak and during the ongoing pandemic. Such information will be valuable to policymakers and stakeholders to design and implement measures to protect those age groups from obesity and its destructive consequences. 

## 2. Materials and Methods

### 2.1. Study Design

A literature search was conducted up to 10 November 2020. Various terms were combined, such as “obesity”, “weight gain”, “overweight”, “obese” and “fat”. References of eligible studies and relevant reviews were also searched, in a snowballing technique.

### 2.2. Inclusion Criteria

All articles that examined obesity or weight gain in children, adolescents and young adults during the COVID-19 pandemic were considered eligible for this review. Regarding study design, cohort studies, cross-sectional studies and case-control studies were considered eligible. There was no language, gender or other demographic restriction. In some studies, the age range included children, adolescents and young adults in a considerable percentage, along with adults (age admixture). Dietary habits or weight gain could be reported by children and adolescents or by parents/carers. Three authors (E.P., E.K. and A.S.) working independently to each other in pairs performed the selection of studies.

### 2.3. Data Extraction and Analysis

A piloted data extraction form was used to extract data from eligible articles, which were reviewed simultaneously and independently from three reviewers (E.P., E.K. and A.S.). The following data were extracted for each study: title of the article, name of first author and year of publication, region/country where the survey was conducted, language, study period, study design, sample size, age range, selection of sample, ascertainment and/or association with COVID-19 epidemic, statistical analysis and main findings about children and adolescent obesity. Any disagreement was resolved by discussion through reviewers and team consensus. 

### 2.4. Quality Assessment

During the screening process, three independent reviewers (E.K., K.P. and A.S.) performed the quality assessment, evaluating the risk of bias in eligible studies, through the Newcastle-Ottawa Scale for cross-sectional studies [11] and cohort studies [12].

## 3. Results

### 3.1. Selection of Studies

From the research in databases, 7171 publications were retrieved. Of them, 850 were duplicates and 5566 were excluded based on title and abstract as irrelevant. More than 59 were deemed reviews, and finally, 15 articles were considered eligible, after full text assessment. Among them, two presented data from various countries (Spain, Italy, Brazil, Colombia, and Chile) [13,14], three referred to the US in general [15,16,17] and one included hot spots of New York, New Jersey, Massachusetts, Maryland, Alabama, Colorado, Connecticut, Pennsylvania, Michigan, Louisiana, Georgia, Illinois, Indiana and District of Columbia [18]. Six were performed in European countries; namely, two retrieved data from Italy [19,20], two from Spain [21,22], one from France [23] and one from Poland [24]; two studies were conducted in China [25,26], and one in Palestine [27]. Most of them (*n* = 8) were cross-sectional, and the other seven were cohort studies, while the majority were conducted in reference to the COVID-19 lockdown (*n* = 9), and some referred to the COVID-19 pandemic (*n* = 6) (Table 1).

From the 15 studies, 9 identified 17,028,111 children, adolescents and young adults from 5 to 25 years old [13,14,15,17,18,20,25,26,27], where the other 5 were considered studies with age admixture (*n* = 20,521) [19,21,22,23,24], and one study included parents with children 5–18 years old (*n* = 584) [16]. 

### 3.2. Weight Gain

According to several studies, during the COVID-19 era, children, adolescents and young adults have increased their food intake and gained weight [16,17,18,21,22,24,26,27]. Specifically, 41.7% of adolescents in Palestine reported gaining weight due to an increase in consumption of fried foods, sweets, sugar-added drinks and dairy products during the lockdown [27]. Worse financial status of the family was associated with more weight gain, mostly due to stress, staying at home, distancing from family and friends, frequent cooking and parents’ concern about COVID-19’s impact [16,27]. Similar findings from China report an increase in youths’ BMI, in all groups (high school, undergraduate and graduate students) [26]. Furthermore, in Poland an increase in BMI was associated with a reduction in vegetable, fruit and legume intake, leading to weight gain (almost 30%) [24].

The higher prevalence of obesity (15% more), among 17 million adolescents, in a cohort study conducted in the US was found in states of Louisiana, Michigan, Indiana, Pennsylvania, Georgia, Alabama, Maryland and New Jersey hot spots [18], while girls, non- Hispanic whites and Asians were at lower risk for obesity due to COVID-19’s impact than boys, non-Hispanic blacks and Hispanics in general [17]. Findings from Spain were mixed, where more than 50% declared no change in their weight, but 25% claimed that their weight was elevated due to depressive symptoms [21]. Obese subjects reported a higher decrease in weight (−3.2 kg) than patients with Anorexia Nervosa (AN), while there was no change in weight for patients with Bulimia Nervosa (BN) and Other Specified Feeding or Eating Disorder (OSFED) [22] (Table 1).

### 3.3. Changes in Dietary Behaviors

The COVID-19 pandemic has caused changes in everyday life, including dietary behaviors. During the lockdown, the number of meals has increased, with potato, meat and sugary drinks being consumed more often among males than females [20]. Compared to the week before the lockdown, females and adolescents from Spain, Brazil and Chile consumed more vegetables and fruits, while adolescents with mothers of higher education ate more vegetables and fruits as well [13]. Among adolescents, the consumption of fried food and sweets increased up to 20.7% during the lockdown and was associated with higher BMI and younger age [13,19]. A moderate increase was noted in daily intake of caloric or salty food (24%) in females with younger age, partner, a residence and with a history of psychiatric disorder [23]. Parents were more concerned about the food intake of their children, with restricting measures and pressure for healthier and nutritious options, to be more frequent in controlling their food [16] (Table 1).

### 3.4. Physical Activity

The restrictions imposed by lockdown include not only social distancing but also reducing movements out of the house. Thus, physical activity was limited to what was absolutely necessary. Parents reported that children’s physical activity (PA) decreased, whereas sedentary behavior (SB) increased [15]. The percentage of children who participated in team sports was 10.4%, 28.9% practiced activity lessons (dance, yoga, etc.) and 2.4% followed gym influencers through remote or streaming services, either inside the house or in the garage/garden [15]. Adolescents from Latin America were less active during quarantine, while adolescents with highly educated mothers were less active as well [13]. Brazil and Chile presented high inactivity during the lockdown (93%) and general inactivity among adolescents in this period (79.5%) [13]. Furthermore, weight changes were associated with limited PA, either vigorous or moderate [21], or no PA during the lockdown at all [28] (Table 1).

### 3.5. Risk of Bias

Half of the studies were cross-sectional studies (*n* = 8), and the majority of them scored high (*n* = 5) in the Newcastle–Ottawa scale, while the others scored either medium (*n* = 2) or low (*n* = 1). In some cases, the selection of the sample was detailed, but in three of them, the non-responder rate was not justified. Due to COVID-19 restrictions, the ascertainment of exposure was implemented through online questionnaires and thus were not always validated. In all of the studies, the control of confounding factors was conducted through appropriate statistical analysis, but the assessment of the outcome suffered mainly from self-reporting, due to COVID-19 social distancing. Furthermore, the seven cohort studies provided good (*n* = 3) and some fair quality (*n* = 4) and only one poor quality (Table S1).

## 4. Discussion

According to the findings of the present review, changes in dietary habits were observed; the number of meals was increased, as well as the consumption of fried food and sweets (up to 20.7%) in a multinational study [13]. Additionally, 41.7% of the adolescents reported weight gain, and half of them stated a rise in their meals and food intake in Palestine [27]. A similar increase was reported in a Spanish study as well, as about 25% declared a weight increase [21], while an Italian study associated the increased consumption of junk food with elevated BMI [19]. As a result of the above-mentioned changes, higher obesity rates were recorded during the pandemic, with the prevalence being increased more than 15% in many US states [18]. During the lockdown, BMI increased significantly in adolescents and young adults (15–17 years) in China, while the prevalence of obesity rose from 10.5% to 12.9% (*p* < 0.001) in these age groups [26].

In 2019, 38.2 million children under the age of 5 years were overweight or obese (almost half of them lived in Asia), and over 340 million children and adolescents aged 5–19 were overweight or obese in 2016. The prevalence of overweight and obesity among children and adolescents aged 5–19 has risen significantly from just 4% in 1975 to more than 18% in 2016. Although obesity and overweight were considered to be a problem in high-income countries, there is also a rapid growth in low- and middle-income countries [28]. In the United States, in 2017–2018, almost 18.5% (13.7 million) of children aged 2–19 years were obese. Non-Hispanic blacks and Hispanics seem to have a higher prevalence of childhood obesity than non-Hispanic whites and Asians; nevertheless, as projection models in the future were used to figure out the increase in childhood obesity, precaution is needed to make assumptions [17]. 

During the COVID-19 pandemic, prolonged school closures were compulsory to reduce infection rates [29]. However, this was a measure that disrupted the daily routine of children attending remote lessons [30], restricting their regular, physical, extracurricular and outdoor activity as public places were closed. The resultant reduction in energy expenditure was a factor associated with an increased risk of childhood obesity [17]. Moreover, excessive screen time was related to sedentary behavior and snacking, which are also associated with obesity, high blood pressure and insulin resistance [31].

Children who rely on school-based health and mental health care, children from food insecure families, obese children, children at risk of abuse and neglect and homeless children are the most vulnerable to school closures [30]. Food insecurity might generate malnutrition, which has progressively been recognized also as a consequence of overnutrition. There may be an interaction between food insecurity, malnutrition and obesity, contributing to poor health and premature death [4]. For families with limited purchasing access to nutrient-dense foods (e.g., fresh fruits and vegetables), the ‘’per calorie’’ cost is higher than calorie-dense junk foods [32]. According to the present review, during the COVID-19 pandemic, the percentage of families reporting difficulty accessing food in the US increased by 20% due to financial reasons, anxiety, difficulty of access as stores were closed and fear of transmitting the virus [16]. The closure of schools increased insecurity as many families relied on the meals they offered. About one third of families preferred to buy high-calorie products, snacks, sweets, desserts and sugary drinks, while an increase of 47% was recorded in the consumption of canned food [16]. At the same time, an increase was observed during the pandemic in the use of food as a “reward” by their parents but also concerning the pressure from parents on their children to eat [16].

School closures disrupted nutrition assistance programs, such as the National School Lunch Program, which enabled all school children in the United States to receive a nutritious lunch every school day. Many states had to reverse the grab-and-go model, which assisted all students to pick up their breakfast and lunch boxes. However, there have been reports about school food service workers having contracted coronavirus disease [18].

Taking into consideration the alarming increase of weight gain in children and adolescents during the pandemics, the American Academy of Pediatrics recommends the assessment of BMI in every pediatric visit and to provide counseling concerning healthy diet and physical activity. Additionally, at-risk children and adolescents should be identified through the assessment of nutrition, sedentary behavior, sleep and physical activity, review of systems, and physical and family history. Return to sports and re-entry at schools are also recommended, depending on each country’s regulations. [33].

The main limitation of the present review was that data derived in many cases from online questionnaires, compromising the validity of results. Additionally, the existing restrictions (lockdown, school closures) and timeline were not always clarified. Most of the cases concerned adolescent and young adults, while studies about younger children were limited. On the other hand, this is the first review concerning weight gain and obesity rates exclusively in these age groups (children, adolescents and young adults) during the COVID-19 pandemic and providing interesting and unique results about these subjects.

## 5. Conclusions

School closures, along with other COVID-19 restrictions, have disrupted the everyday routine of children, adolescents and even young adults, leading to changes in their eating behaviors and physical activities. Unhealthy choices concerning everyday meals seem to have prevailed during the pandemic. Many countries such as China and the USA have already reported an increase in obesity rates in children and adolescents, confirming the existing fears. Another consequence of the pandemic, food insecurity, meaning limited access to food, may also lead to malnutrition and obesity. As the pandemic is still ongoing, in order to protect the above-mentioned age groups, health care providers should highlight the risk of obesity and provide prevention strategies, including parental participation. Worldwide policies, guidelines and precautionary measures should ideally be established.

## Figures and Tables

**Table 1:** Description of studies examining obesity in children and adolescents.

First Author (Year)	Region, Country		Study Period	Study Design	Sample	Sample Size	Age Range	Selection of Sample	Ascertainment and/or Association with the COVID-19 Pandemic	Statistical Analysis	Main Findings
Di Renzo, L. et al. (2020) [19]	All Italian Regions		5 to 24 April 2020	Cross-sectional	The Italian population	3533	12–86	A survey questionnaire of 43 questions was distributed through social networks such as Twitter, Facebook and Instagram, institutional mailing lists and the “PATTO in Cucina Magazine” website.	COVID-19 lockdown	The Shapiro–Wilk test was performed to evaluate variables distribution. The Spearman correlation coefficient was used for correlations between continuous variables and Chi square test for categorical variables. McNeman analysis was used to investigate the difference between categorical variables pre and during the COVID-19 emergency and Mann–Whitney U and Kruskal–Wallis tests for continuous variables. Results were significant for *p* value < 0.05. Statistical analysis was performed using SPSS ver. 21.0 (IBM, Chicago, IL, USA).	South and Islands had a population with higher BMI when compared to North and Center Italy (*p* = 0.007, *p* = 0.008; respectively), 674 participants (19.1%) were students. BMI and age were positively and inversely linked to the increased appetite and night snacks, respectively (OR = 1.073, *p* < 0.001; OR = 0.972, *p* < 0.001). Increase junk food consumption was related to higher BMI and lower age (OR = 1.025, *p* = 0.005; OR = 0.979, *p* < 0.001), while increased risk of junk food intake was also associated with enhanced appetite and after-dinner hunger (OR = 4.044, *p* < 0.001; OR = 1.558, *p* < 0.001). No association was observed between the increase of healthy food intake, BMI and age (*p* = 0.381, *p* = 0.053). A reduced appetite was related to a major consumption of healthy foods (OR = 1.718, *p* < 0.001) the population group aged 18–30 years resulted to have a higher MEDAS score comparing to younger and elder population (*p* < 0.001; *p* < 0.001, respectively)
Ruiz-Roso, M.B. et al. (2020) [13]	Several regions of Spain, Italy, Brazil, Colombia and Chile	English	17 April to 25 May, 2020	Cross-sectional	Adolescents	820	10–19	Invitation to participate through social media (Facebook, Instagram and WhatsApp) or by e-mail	COVID-19 confinement	Paired two-way Student’s *t*-test, two-way ANOVA, chi-square test	Forty-three percent of adolescents consumed vegetables every day during confinement versus 35.2% who did it before. During confinement, 64% of adolescents consumed fast food at least once a week compared to 44.6% before confinement. While 14% of adolescents consumed sweet food every day before COVID-19, there was an increase to 20.7%. The highest rates of adherence to the weekly food intake recommendation were observed in females adolescents living in Europe with a higher maternal education. Females significantly increased their vegetable and fruit intake (*p* < 0.0001) compared to before and consumed significantly more fruits and vegetables than boys. Males, on the other hand, did not change their average fruit consumption and presented an increase in vegetable consumption (*p* = 0.0007), and processed meat intake (*p* = 0.0182). Adolescents under the age of 14 significantly increased the average consumption of fried and sweet foods (*p* = 0.0025 and *p* = 0.0386), while those over 14 increased vegetable and fruit intake. There was a dramatic increase in sweet food consumption in those over 17 years of age. Increase in vegetable consumption during confinement was recorded in adolescents from Spain, Brazil and Chile, but not Italy and Colombia, while adolescents from Brazil had a higher average legume intake. Colombian adolescents significantly decreased sugar-sweetened beverage intakes, while Chileans significantly increased fried food intake (*p* < 0.0001).
Pietrobelli, A. et al. (2020) [20]	Verona, Italy.	English	From pre-lockdown (May–July 2019) to lockdown (March–April 2020)	Longitudinal observational study	Children and adolescents with obesity	41	13.0 (Mean) ± 3.1 (SD) years (range, 6–18) years	Longitudinal observational OBELIX Study approved by the hospital Institutional Review Board (Protocol: 5384, 29 January 2019)	Pre-peri lockdown period	Paired *t*-tests, Pearson correlation analyses, Independent two-sample *t*-tests was declared if a two-sided	During the lockdown, the number of meals per day increased (*p* < 0.001), more in males than in females (*p* = 0.028), with no changes in vegetable and fruit intake (marginal significance, *p* = 0.055). Potato chips, red meat and sugary drink intakes all increased significantly, (p value range from 0.005 to <0.001). Sleep time and screen time increased significantly, (*p* = 0.003 and *p* < 0.001 respectively), while sports time decreased significantly (*p* = 0.003).
Ruiz-Roso, M.B. et al. (2020) [14]	Countries in Europe (Italy and Spain) and Latin America (Brazil, Chile and Colombia)	English	17 April to 20 May, 2020	Cross-sectional	Adolescents, mostly females	726	10–19	Structured questionnaire created in Google Forms (Google LLC, Menlo Park, CA, USA) in an anonymous electronic survey, also known as an e-survey or web survey	The periods of lockdown varied according to the evaluated countries, but they all occurred in March	Chi-squared tests were performed. Variables with *p* < 0.10 were included in a multinomial logistic regression model. Univariate logistic regression	About 73.0% of adolescents were considered physically inactive before social isolation compared to 79.5% during the lockdown. The highest frequencies of inactive adolescents during isolation were observed in Brazil and Chile. Inactivity increased from 40.9% to 93% during the evaluated period (*p* < 0.001) in Brazil. In Latin America, adolescents presented an odds ratio (OR) of 2.98 (CI 95% 1.80–4.94) of being physically inactive during quarantine, and living in this region was connected to habitual ultra-processed foods consumption (OR 1.58; *p* = 0.007. Boys were more active (OR 2.22 (CI 95% 1.28–3.86)) while adolescents with highly educated mothers were less active (OR 0.40 (CI 95% 0.20–0.84)).
Dunton, G.F., Do, B., Wang, S.D. (2020) [15]	US.	English	25 April to 16 May, 2020 and a second online survey was scheduled to occur within 6–12 months	Cohort	Children	211	5–13	Respondents were electronically invited through various social media platforms (e.g., Facebook, Twitter) and university-based email list of students, faculty, and staff	COVID-19 pandemic	Chi-square and independent samples *t*-tests, multiple linear regression analyses, ordinal logistic regression models	Parents perceived that children’s physical activity had decreased whereas children’s sedentary behavior had increased between the pre-COVID-19 period (February 2020) and the early COVID-19 period (April–May 2020). Performance of physical activity at home or in the garage (OR = 2.49, 95% CI [1.35, 4.60], Wald = 8.593, *p* = 0.003) and on sidewalks and roads in their neighborhood (OR = 1.92, 95% CI [1.04, 4.60], Wald = 4.28, *p* = 0.038) increased from the during the early COVID-19 period. Additionally, in this period, 10.4% of children participated in team sports training sessions or practice through remote or streaming services, 28.9% participated in activity classes or lessons through remote or streaming services and 2.4% participated in remote or streaming classes or sessions provided by a health club or gym. Older children (ages 9–13) vs. younger children (ages 5–8) were more likely to participate in team sports training sessions or practice through remote or streaming services (OR = 5.40, 95% CI [1.70, 17.15], Wald = 8.19, *p* = 0.004).
An, R. (2020) [17]	US	English	April 2020 to March 2021	Cohort	Children	15,631	5–6	Early childhood longitudinal study, Kindergarten Class of 2010–2011 (ECLS-K:2011)	COVID-19 pandemic	Restricted cubic spline regressions, microsimulation model	An increase in the mean body mass index z-scores and in childhood obesity prevalence was observed during COVID-19. The impact on childhood obesity during this period was modestly smaller in girls and non-Hispanic whites and Asians than boys and non-Hispanic blacks and Hispanics.
Fernández-Aranda, F. et al. (2020) [22]	Barcelona, Spain	English	June and July 2020	Cross-sectional	Patients	121 participants (87 Eating Disorder patients and 34 patients with obesity)	13–77 (mean = 33.7, SD = 15.8)	Patients from six different child–adolescent and adult units from the Barcelona region	COVID-19 pandemic	Confirmatory factor analyses (CFA), paired-sample *t*-tests for interval scaled variables, and the McNemar test for categorical measures	Significant decreases were recorded to anorexia nervosa patients after the confinement considering the impact on eating symptoms, changes in eating style and changes in emotion regulation. A significant decrease in weight was also observed in obese patients concerning BMI and changes in the eating style. In this group (obese patients) the highest change in weight (with a significant decrease of 3.2 kg, compared to an increase of nearly 1 kg for anorexia nervosa patients) was observed.
Adams, E.L. et al. (2020) [16]	US	English	30 April to 23 May, 2020	Cross-sectional	Parents	584	18 years of age or older and had at least 1 child between 5–18 years of age	Social media advertisements on Facebook, snowball technique by circulating the survey link via email, on listservs	COVID-19 pandemic	Chi-square test, paired sample *t*-tests, univariate regression models	A 20% increase during COVID-19 was reported considering families with low food security (*p* < 0.01). The amount of high-calorie snack foods, desserts/sweets and fresh foods was increased in 1/3 of families, while a 47% increase was recorded on non-perishable processed foods. Use of restriction, pressure to eat and concern about child overweight increased during COVID-19, with a greater increase in pressure to eat for parents with food insecurity compared to food secure parents (*p* < 0.05)
Fernandez-Rio, J. et al. (2020) [21]	Spain	English	March to April 2020	Cross-sectional	Spanish citizens	4379	16–84	Nonprobability snowball sampling was used to recruit participants through a web link distributed via e-mail, WhatsApp, Twitter.	COVID-19 lockdown	Chi-square, multinomial logistic regression analyses, restricted cubic splines models	No weight changes were reported in 52.88%, while 25.82% reported weight increase and 21.27% weight decrease. Greater weight variability was recorded on males, young and obese individuals. Depressive symptoms were associated with larger weight changes.
Jia, P. et al. (2020) [25]	China	English	May 9 to 12, 2020	Retrospective cohort study	Youths in China	10.082	15–28	COVID-19 Impact on Lifestyle Change Survey (COINLICS)	COVID-19 lockdown	Paired *t*-tests (or χ^2^ tests for categorical variables)	A significant change was recorded in the participants’ diet. Significant decreases were recorded in the intake of rice, fresh vegetables, meat, fresh fruit, poultry, dairy products and soybean products during the COVID-19 lockdown. Females consumed more fresh vegetables, fruit and rice and less poultry, meat, soybean and dairy products. On the other hand, significant increases were reported in the consumption of wheat products and preserved vegetables, with males consuming these foods more commonly. A decrease was observed in the frequency of sugar-sweetened beverage consumption and an increase in the frequency of tea drinking.
Allabadi et al. (2020) [27]	West Bank, Palestine	English	April 24 to 27, 2020	Cohort	Adolescents	600	10–19	Of the sample, 65.7% were chosen using randomly generated phone numbers and 34.3% were chosen using snowball sampling	COVID-19 lockdown	Bivariate analyses, Chi-Squared test or Student’s *t*-test, Multinomial logistic regression	The percentage of adolescents who gained weight was 41.7%, while 50.0% of adolescents reported an increase in their food intake. Weight gain seemed to be more likely among those with increased food intake (*p* < 0.001) and those who declare more sugar-added drinks (*p* < 0.001), fried foods (*p* = 0.07) and sweets (*p* < 0.001) consumption. Additionally, weight gain was more common among those with no physical activity during the lockdown (*p* = 0.05), those with increased screen time during the lockdown (*p* = 0.001) and those who reported a worsening family financial situation (*p* < 0.001). Moreover, increased weight and increased food intake during the lockdown were also associated with the following factors: staying at home, distance learning, not going to work, financial situation and distance from family and friends.
Yang, S. et al. (2020) [26]	China	English	23 December, 2019 to 23 January, 2020 and 24 Januar to 23 February, 2020	Cohort	Youths in China	10.082	17–25	COVID-19 Impact on Lifestyle Change Survey (COINLICS)	COVID-19 lockdown	Paired t-tests (or χ^2^ tests for categorical variables) Non-Parametric methods to test the significance of differences in activity patterns among groups (Mann–Whitney U test and Kruskal–Wallis	Within the month during COVID-19 lockdown, BMI significantly increased in overall youths (21.8–22.6, *p* < 0.001) and in all subgroups: high school (22.7–23.8, *p* < 0.001), undergraduate (21.4 to 22.2, *p* < 0.001) and graduate students (21.4–22.3, *p* < 0.001). Moreover, the prevalence of overweight/obesity significantly increased generally (21.3–25.1%, *p* < 0.001) and in high school (26.6–30.3%, *p* < 0.01) and undergraduate students (19.1–22.8%, *p* < 0.001); obesity prevalence also significantly increased generally (10.5–12.9%, *p* < 0.001) and in high school (16.0–18.8%, *p* < 0.01) and undergraduate students (8.5–10.7%, *p* < 0.001)
Dutta, M. (2020) [18]	USA	English	11 April to 20 May, 2020	Cohort	Schoolage children	17 million	10–17	Data collected from “COVID-19 hot spots” (any state with 15% or more confirmed COVID-19 cases out of total tests”) compared with data on obesity for children of same ages collected from each state level at 2017–2018	COVID-19 pandemic-school closures	-	Many of the COVID-19 hot spots, including the states of Louisiana, Michigan, Indiana, Pennsylvania, Georgia, Alabama, Maryland and New Jersey had a higher prevalence of obesity among the studied population at 15% or more.
Sidor, A. and Rzymski, R. (2020) [24]	Poland	English	17 April to 1 May, 2020	Cross-sectional	Polish adults	1097	18–45	Online survey	COVID-19 lockdown	Mann–Whitney U test or Kruskal–Wallis analysis of variance (ANOVA) with Dunn’s post-hoc method, Spearman’s correlation coefficient	Almost 30% reported weight gain (mean ± SD 3.0 ± 1.6 kg, mostly overweight, obese, and older subjects), and weight loss was recorded only in 18% (−2.9 ± 1.5 kg). Less frequent consumption of vegetables, fruit and legumes during quarantine and higher adherence to meat, dairy and fast foods was associated with increased BMI.
Rolland et al. (2020) [23]	France	English	25 March to 30 March, 2020	Cross-sectional	French citizens over 16	11.391	16–75	Online survey on the internet using social media (i.e., Twitter, LinkedIn, and Facebook) and national media	COVID-19 lockdown	Logistic regression models, raw odds ratios (ORs) and adjusted odds ratios (aORs) are provided with their 95% confidence intervals	The percentage of participants who reported no changes in their average daily intake of caloric/salty food was 57.14% (6510/11,391), whereas 24.9% (2836/11,931) declared a moderate increase and 3.49% (397/11,931) an increase in a difficult-to-control manner. The percentage who reported a reduction in their intake without craving was 7.67% (874/11,391), and 1.35% (154/11,391) with craving.

## Data Availability

Data is contained within the article.

## Supplementary Materials

The following are available online at https://www.mdpi.com/2227-9067/8/2/135/s1, Table S1: Newcastle Ottawa Scale for cross-sectional and cohort studies.
