# Supplementary material for: Obesity in Children and Adolescents during COVID-19 Pandemic
Source: Children (Basel). 2021 Feb 12;8(2):135. doi: 10.3390/children8020135 (PMC7918914; doi:10.3390/children8020135)
Supplement: Supplementary file 1 [file children-08-00135-s001.pdf]

Table S1. Newcastle Ottawa Scale for cross-sectional and cohort studies.

Cross-sectional studies								
	**Selection**				**Comparability**	**Outcome**		**Total**
**First author (year)**	**Representativeness of the sample**	**Sample size**	**Non-respondents**	**Ascertainment of exposure**	**The subjects in different outcome groups are comparable, based on the study design or analysis. Confounding factors are controlled.**	**Assessment of outcome**	**Statistical test**	
Di Renzo L et al. (2020)	*	*	*	**	**	*	*	9/10
	Truly representative	3,533 Italian citizens, 674 students among them	A total of *n* = 4.500 participants started the survey, *n* = 3,533 considered valid	The EHLC-COVID19 questionnaire was specifically built by using Google Form by the Section of Clinical Nutrition and Nutrigenomic, Department of Biomedicine and Prevention of the University of Rome Tor Vergata and the MEDAS questionnaire to assess adherence to the MD.	Residence region, age, gender, BMI, current employment	Self report	The Shapiro–Wilk test, the Spearman correlation coefficient, Chi square test, McNeman analysis, Mann–Whitney U and Kruskal–Wallis tests, binary and multinomial logistic regression analyses	
Ruiz-Rosso MB et al. (2020)	*	*	*	**	**	*	*	9/10

	Truly representative	820 adolescents	From a total of $n = 828$ eligible in the final sample was $n = 820$	Dietary practices were evaluated using a standardized adolescent questionnaire, the National School Health Survey–PeNSE; Pesquisa Nacional de Saúde do Escolar, which was slightly modified	Age, gender, country, maternal education, number of family members, watching TV during mealtimes	Self report	Paired two-way Student's t-test,ntwo-way ANOVA. A significance level of $p < 0.05$ was applied to all statistical analyses.	
Ruiz-Rosso MB et al. (2020) 32751721	*	*	*	**	**	*	*	9/10
	Truly representative	726 adolescents	Among those, eight (1.1%) declined to participate.	To assess the level of physical activity, the International Physical Activity Questionnaire (IPAQ) was used. For ultra-processed food consumption, was considered the assessment of the weekly consumption of ultra-processed foods, so named according to the NOVA classification	Country, sex, physical activity status, ultra-processed food consumption, maternal education, number of residents at home	Self report	Chi-squared tests, multinomial logistic regression model and univariate logistic regression were used	
Fernández-Aranda F et al. (2020)	*	*	-	*	* *	* *	*	8/10
	Truly representative	121 participants (87 ED patients and 34 patients with obesity)	No description	COVID Isolation Eating Scale (CIES), a newly created scale for measuring the impact of confinement	Sex, age, eating disordes diagnosis, obesity	Record linkage, self report	Confirmatory factor analyses, paired-sample t-tests for interval scaled variables, and the Mc Nemar test for categorical measures	
Adams EL et al. (2020)	*	*	*	* *	* *	*	*	9/10

	Somewhat represantative	584 parents	A total of *n* = 1,342 parents started the survey, *n* = 584 considered valid	6-item United States Department of Agriculture Household Food Security Module, Child Feeding Questionnaire (CFQ)	Parents age, children age, race, marital status	Self report	Chi-square test, Paired sample t-tests, univariate regression models	
Fernandez-Rio J et al. (2020)	*	*	-	*	* *	*	*	7/10
	Snowball sampling	4379 Spanish citizens	No description	On-line questionnaire	Sex, age, BMI, depressive symptoms, optimism	Self report	Chi-square test, multinomial logistic regression analyses, restricted cubic splines models	
Sidor A and Rzymski R (2020)	-	*	-	*	**	*	*	6/10
	Specific group of subjects	1097 Polish citizens	No description	Online self-designed, structured questionnaire	Age, gender, weight, bmi, occupation	Self report	Mann–Whitney U test or Kruskal–Wallis analysis of variance (ANOVA) with Dunn's post-hoc method	
Rolland et al. (2020)	-	*	*	*	* *	*	*	7/10
	Convenience sampling method	11.391 French citizens	Among the 20,235 participants who started the questionnaire, 11,742 (58.0%) completed it. After excluding inoperable questionnaires and respondents from countries other than France, 11,391/20,235 questionnaires (56.3%) were included	Online questionnaire	Age, gender, marital status, employment status, educational level	Self report	Logistic regression models, raw odds ratios (ORs) and adjusted odds ratios (aORs) are provided with their 95% confidence intervals	

			in the analyses					

Cohort studies								
**Selection**					**Comparability**	**Outcome**			**Total**
**First author (year)**	**Representativeness of the exposed cohort**	**Selection of the non-exposed co-hort**	**Ascertainment of exposure**	**Demonstration that outcome of interest was not present at start of study**	**Comparability of co-horts on the basis of the design or analysis controlled for con-founders**	**Assessment of outcome**	**Was follow-up long enough for outcomes to occur**	**Adequacy of follow-up of cohorts**	
Pietrobelli A et al. (2020)	*	*	*	-	*	-	*	*	good quality
	Children and adoles-cents with obesity (range, 6–18) years	Same com-munity	Structured inter-view	No	Sex, age, living area	Telephone interview	Yes	Complete follow up	
Dunton GF, Do B, Wang SD (2020)	*	*	*	*	**	-	*	*	good quality
	Children 5 to 13 years, with an aver-age age of 8.71 years (SD = 2.58)	Same com-munity	Structure inter-view, self report	Pre-COVID-period (February 20,200 and early-COVID-19 period (April–May 2020)	Parental age,sex, marital status, work status, annual household income, child age, sex, ehnic-ity, race	Self report	Yes	Complete follow up	

An R (2020)	*	*	*	-	* *	*	*	*	good quality
	Children 5-6 years in US	Same community	ECLS-K:2011	No	Sex, racial/ethnic differences, physical acivity	Record linkage	Yes	Complete follow up	
Jia P et al. (2020)	*	*	*	-	* *	-	-	*	fair quality
	Youths in China	Same community	COVID-19 Impact on Lifestyle Change Survey (COINLICS)	No	Sex, age, ethnic group, living areas	Self report	No	Complete follow up	
Allabadi et al. (2020)	*	*	*	-	* *	-	-	*	fair quality
	Ain Palestine	Same community	Structure interview, self report	No	Sex, age, marital status, education	Self report	No	Complete follow up	
Yang S. et al. (2020)	*	*	*	-	* *	-	-	*	fair quality
	Youths in China	Same community	COVID-19 Impact on Lifestyle Change Survey (COINLICS)	No	Sex, age, education, ethnic group, living areas	Self report	No	Complete follow up	
Dutta M (2020)	*	*	*	-	*	*	-	-	poor quality
	Schoolage children USA	Same community	COVID-19 tracking project	No	Age	Record linkage	No	No statement	
